# Hydrogels in Peri-Implant Regeneration: Strategies for Modulating Tissue Healing

**DOI:** 10.3390/pharmaceutics17091105

**Published:** 2025-08-25

**Authors:** Paula Buzo Frigério, Nathália Dantas Duarte, Mateus Meister Koury, Felipe de Souza Duarte, Roberta Okamoto, Daniela Vieira Buchaim, Carlos Henrique Bertoni Reis, William Saranholi da Silva, Lívia Maluf Menegazzo Bueno, Marcio Cristino Raphael, Rogerio Leone Buchaim, João Paulo Mardegan Issa

**Affiliations:** 1 Department of Diagnosis and Surgery, Aracatuba School of Dentistry (FOA-UNESP), São Paulo State University, Aracatuba 16015-050, Brazil; paula.frigerio@unesp.br (P.B.F.); nd.duarte@unesp.br (N.D.D.); fs.duarte@unesp.br (F.d.S.D.); 2 Central Unit, Department of Biomedicine, Barão de Mauá University Center, Ribeirão Preto 14090-180, Brazil; mateuskoury@gmail.com; 3 Department of Basic Sciences, Aracatuba School of Dentistry (FOA-UNESP), São Paulo State University, Araçatuba 16015-050, Brazil; roberta.okamoto@unesp.br; 4 Graduate Program in Anatomy of Domestic and Wild Animals, Faculty of Veterinary Medicine and Animal Science, University of São Paulo (FMVZ-USP), São Paulo 05508-270, Brazil; danibuchaim@alumni.usp.br (D.V.B.); rogerio@fob.usp.br (R.L.B.); 5 Medical, Pharmacy and Dentistry School, University Center of Adamantina (FAI), Adamantina 17800-000, Brazil; likamaluf@usp.br (L.M.M.B.); marcio.raphael@fai.com.br (M.C.R.J.); 6 Department of Postgraduate, Dentistry School, Faculty of the Midwest Paulista (FACOP), Piratininga 17499-010, Brazil; 7 Beneficent Hospital (HBU), University of Marilia (UNIMAR), Marilia 17525-160, Brazil; dr.carloshenriquereis@usp.br; 8 Dentistry School, University of Marilia (UNIMAR), Marilia 17525-902, Brazil; williansaranholi@unimar.br; 9 Department of Biological Sciences, Bauru School of Dentistry (FOB-USP), University of São Paulo, Bauru 17012-901, Brazil; 10 Department of Basic and Oral Biology, Ribeirão Preto School of Dentistry (FORP-USP), University of São Paulo, Ribeirão Preto 14040-904, Brazil

**Keywords:** hydrogels, peri-implant, bone repair, bone regeneration, biomaterials

## Abstract

**Background/Objectives**: Hydrogels have emerged as strategic biomaterials in bone tissue engineering, especially in the peri-implant context, due to their high biocompatibility, water retention capacity, three-dimensional defect filling, and ability to mimic the extracellular matrix. These properties allow physical support for regeneration and the incorporation and controlled release of bioactive, immunomodulatory, and osteoinductive agents. **Methods**: This narrative review aimed to summarize recent advances in developing and applying hydrogels for the repair of peri-implant bone defects. The selection of studies was performed in PubMed, Web of Science, and EBSCO databases, covering the period from 2010 to 2025. Thus, 14 preclinical and clinical studies were included in this review. **Results and Conclusions**: Hydrogels show great potential for peri-implant bone regeneration due to their biocompatibility and ability to deliver bioactive agents. While preclinical results are promising, clinical validation remains limited. Further studies are needed to confirm their efficacy and ensure the safe translation of these findings into clinical practice.

## 1. Introduction

The regeneration of bone defects remains one of the main challenges of regenerative medicine, especially in highly complex environments such as those surrounding dental and orthopedic implants [1]. Clinical situations such as extensive fractures, peri-implant bone loss, osteoporosis, and metabolic diseases, such as type 2 diabetes, often limit the spontaneous regeneration capacity of bone tissue and increase the risk of implant osseointegration failures [2,3,4]. It is estimated that between 8 and 10 years after installation, the failure rate of dental implants is around 2.4% [5].

In this scenario, the development of strategies based on biomaterials has been a promising alternative, with emphasis on hydrogels. Hydrogels consist of three-dimensional networks of cross-linked polymers characterized by their ability to retain large volumes of aqueous fluids due to their hydrophilic structure [6,7]. A highly studied characteristic is their biocompatibility, ability to mimic the extracellular matrix (ECM), and enhance the local delivery of bioactive compounds to promote bone regeneration [7,8,9,10,11].

The increasing sophistication of hydrogels has driven efficient advances in bone regeneration, especially in peri-implant contexts, where osseointegration in compromised microenvironments is essential. Formulated from natural, synthetic, or hybrid polymers, these biomaterials can be functionalized with osteoinductive agents, antimicrobials, or therapeutic drugs, modulating changes in the local environment [4,12,13].

Preclinical models demonstrate that the incorporation of substances such as zoledronic acid and bone morphogenetic protein BMP-2 into hydrogels significantly improves implant stability and enhances bone volume and bone–implant contact [14], offering a promising alternative to systemic drug administration, specific side effects, and optimizing peri-implant regeneration [4,15,16,17].

Furthermore, advances in the engineering of injectable hydrogels with in situ polymerization properties enable their application in complex bone defects, offering effective adhesion, three-dimensional filling, and potential for vertical regeneration in critical peri-implant areas [1,18,19]. Photopolymerizable hydrogels, for example, can be used for in situ repairs by minimally invasive optical sources with excellent three-dimensional filling capacity and adaptation in critical areas [20]. Self-regenerative multifunctional hydrogel systems stand out for acting in an integrated manner on intelligence, technology, and regeneration, favoring osseointegration even in adverse conditions, through immune modulation and stimulation of osteogenesis with the use of stem cells and bioactive agents [11,21].

In the dental field, comparative studies demonstrate that polyethylene glycol (PEG)-based hydrogels can be used as scaffolds for guided bone regeneration and as delivery systems for osteoinductive proteins. However, evidence indicates that, when used alone, these hydrogels may present limitations regarding matrix stability in critical defects. The combination with biphasic bone substitutes, such as hydroxyapatite/tricalcium phosphate (HA/TCP), has shown greater clot stability and bone formation in contact with implants. Studies in animal models revealed that the combination of PEG with or without RGD sequence promoted partial bone regeneration in peri-implant dehiscence defects, but with less efficacy when compared to traditional techniques with collagen membrane, which demonstrated superiority in bone formation and bone–implant contact [16,22].

Given the increasing complexity of the challenges in peri-implant bone repair, there is increasing interest in the development of multifunctional hydrogels as smart platforms for tissue regeneration. These biomaterials have stood out for their modulable physicochemical properties, capacity for controlled release of bioactive agents, and dynamic interaction with the bone microenvironment [2,11,13,17].

Therefore, this narrative review aims to present an up-to-date analysis of the most promising approaches involving hydrogels in peri-implant bone regeneration. It will cover the biological foundations of bone regeneration, the classification and composition of hydrogels used in bone repair, and their essential physicochemical properties, as well as their application as controlled systems for biomolecules. In addition, evidence of these therapeutic platforms will be discussed. The integration between biomaterials engineering and bone biology places functionalized hydrogels as strategic tools for development and increasingly effective, safe, and personalized regenerative therapies in implant dentistry.

## 2. Materials and Methods

**Figure 1:**
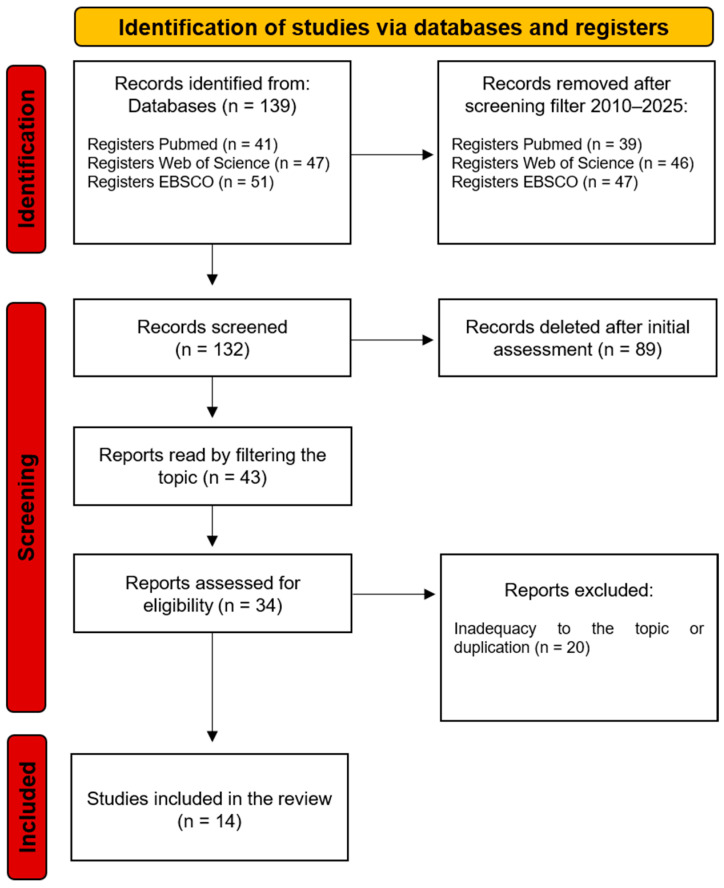
Flowchart of the search and selection process for studies included in the narrative review methodology.

This narrative review was conducted following the guidelines of the Scale for the Assessment of Narrative Review Articles (SANRA) to ensure quality, coherence, and scientific relevance [23]. Literature searches were performed in the electronic databases PubMed, Web of Science, and the EBSCO database aggregator, using the following combined descriptors: “hydrogel” and “peri-implant”. Original articles and published reviews were included, with restrictions on the year of publication, covering the period from 2010 to 2025; however, there were no restrictions on language. In vitro, in vivo, and clinical research publications were considered, prioritizing those of greater scientific and/or clinical relevance. The selection of studies was based on criteria of applicability, technological innovation, biological properties of hydrogels, and potential impact on peri-implant bone regeneration (Figure 1).

As this is a narrative review, no statistical analysis or meta-analysis was performed, and there was no systematic assessment of the risk of bias. The selection of articles prioritized studies with descriptive data, experimental evidence, and translational applicability to peri-implant bone repair guided by the use of hydrogels.

### 2.1. Inclusion Criteria

1. Studies addressing the use of hydrogels applied to peri-implant bone repair, whether in vitro or in vivo models, or clinical trials;
2. Publications in English, Portuguese, or Spanish are available in full text in the PubMed, Web of Science, and EBSCO databases;
3. Articles published with a period restriction, covering the years 2010–2025, up to the time of the search.

### 2.2. Exclusion Criteria

1. Studies that deal with the use of hydrogels in tissues other than bone (e.g., cartilage, skin);
2. Articles focusing exclusively on hydrogels applied to the cure of peri-implantitis or treatment of critical defects in non-peri-implant regions;
3. Articles focusing exclusively on hydrogels applied to aesthetic dentistry, with no relation to peri-implant bone regeneration;
4. Works with duplicate information, poorly supported reviews, or a lack of data relevant to the topic.

## 3. Results

### 3.1. Fundamental Concepts in Bone Regeneration and the Role of Hydrogels

The peri-implant region is composed of anatomical structures essential for the functional stability of dental implants, including the alveolar bone and surrounding soft tissues. Unlike the natural periodontium, the absence of the periodontal ligament at the bone–implant interface modifies the biomechanics of the functional load and alters the local cellular and inflammatory response profile, making the environment more susceptible to degenerative processes [8].

Peri-implant bone tissue is essential for osseointegration and is constantly remodeling, being influenced by mechanical stimuli, inflammation, and the quality of the host tissue. Marginal bone loss, common in late failures, can occur due to occlusal overload, bacterial contamination, poor prosthetic adaptation, or chronic inflammation [16,19].

Because of this, regenerative strategies should consider not only the replacement of lost tissue but also the modulation of the peri-implant microenvironment. Hydrogels stand out as versatile biomaterials due to their ability to conform three-dimensionally to critical defects, support cellular protection, promote angiogenesis, and release bioactive factors in a controlled manner [18,21].

Bone regeneration depends on a complex orchestration between stem cells, growth factors, extracellular matrix, and immune signaling. Injectable hydrogels functionalized with bioactive molecules have demonstrated potential in challenging clinical conditions, such as osteoporosis and type 2 diabetes. For example, sustained-release hydrogels of BMP-2 accelerated bone regeneration in compromised models [3].

The combination of hydroxyapatite nanoparticles (nHA) with zoledronate in vitro and in vivo models promotes peri-implant bone repair, especially in postmenopausal osteoporosis conditions [15]. Deng et al. [2] showed that local release of semaphorin 3A by hydrogels restored osseointegration in diabetic models, promoting osteogenesis.

Li et al. [8] demonstrated that GelMA/SilMA hydrogels with gingival stem cells improved epithelial sealing and induced M2 macrophage protection, creating a favorable environment for healing. Such effects directly support osseointegration and enhance peri-implant stability. A conductive hydrogel responsive to endogenous electric fields was developed that stimulates neural regeneration and increases the release of osteogenic neuropeptides, such as Calcitonin Gene-Related Peptide (CGRP) and Substance P (SP), as developed by Qin et al. [24]. These effects promoted peri-implant bone formation and favored the functional osseointegration of implants.

Therefore, hydrogels emerge as multifunctional platforms that combine mechanical, immunomodulatory, and biological properties. Their application in the peri-implant context goes beyond the simple filling function, acting as intelligent interfaces capable of modulating the regenerative microenvironment in a targeted and effective manner.

### 3.2. Classification of Hydrogels Used in Bone Repair

**Figure 2:**
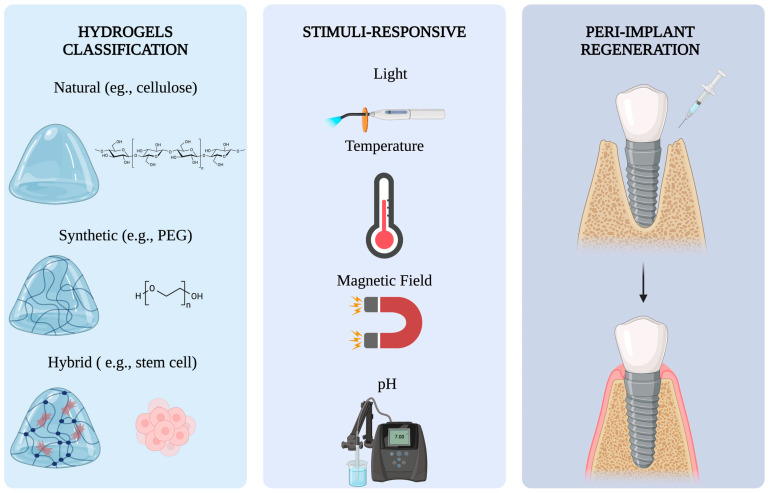
Schematic representation of hydrogel systems applied to peri-implant bone regeneration. Hydrogels can be classified into three types: natural, synthetic, and hybrid, each with unique structural and biological features. These biomaterials can be stimulus-responsive to various stimuli, including light, temperature, magnetic fields, and pH. Their application in peri-implant defects may enhance bone regeneration. Abbreviations: PEG, polyethylene glycol. Created with https://www.biorender.com/ (2 July 2025).

Hydrogels applied in bone tissue engineering can be classified according to their origin: natural (collagen, hyaluronic acid, alginate), synthetic (polyethylene glycol [PEG] or poly(N-isopropylacrylamide) [pNIPAM]) or hybrid (combination of natural and synthetic components), as well as by their response to physiological conditions (responsive to pH, temperature, etc.). In addition, they can be differentiated based on their crosslinking structure, which can be physical (non-covalent interactions) or chemical (permanent or dynamic covalent bonds) (Figure 2).

Among the 14 studies reviewed, hydrogels based on PEG and HA-pNIPAM stand out, often functionalized with osteogenic and osteomodulatory biomolecules, such as bone morphogenetic protein type 2 (BMP-2) and zoledronic acid (ZOL), to promote peri-implant bone regeneration (Table 1 and Table 2).

The works of Jung et al. [22], Thoma et al. [16], and Cha et al. [25] demonstrated the application of PEG as a matrix for combination with bone substitutes or growth factors. Kang et al. [26] used nanoporous sodium titanate hydrogel to evaluate cell viability, while Kettenberger et al. [15] and Kettenberger et al. [27] explored the use of a hyaluronic acid gel incorporated or not with hydroxyapatite nanoparticles (nHA), respectively, functionalized with zoledronic acid (ZOL).

In some cases, as in the studies of Qin et al. [24] and Siverino et al. [3], hydrogels with conductive or thermoresponsive properties were used, capable of responding to bioelectric or physiological stimuli (such as temperature), enabling the controlled release of drugs and the stimulation of neural regeneration and osteogenesis, promoting functional osseointegration.

**Table 1:** Classification of hydrogels based on the analysis of the articles selected for this review.

Origin	Hydrogel Composition	Implant Placement	Preparation	Reference
Natural	HPMC/Si-HPMC	Mandible	HPMC: manual mixing Si-HPMC: constant mixing followed by dialysis; reticulation achieved in Situ via acid buffer	[19]
Synthetic	Hyaluronic acid (AuxiGel™) + ZOL	Femur and proximal tibia	Commercial gel with ZOL	[27]
	PEG + BMP-2	Mandible (alveolar ridge)	Michael addition	[22]
	Hyaluronic acid + PEG-SH4 + rhBMP-2	Mandible	Michael addition reaction	[28]
	PEG + SEMA3A	Femoral condyle	Michael addition	[2]
Hybrid	GMPA(GMP + OHA)	Mandible	Schiff base reaction	[24]
	PEG + SBS + BMP-2 SBS + PEG	Mandible	Michael addition to form PEG, followed by manual mixing to add SBS	[25]
	PEG + RGD + HA/TCP	Maxilla (alveolar ridge)	Michael addition	[16]
	Poly(phosphazene) (IGSR) + BMP-2	Mandible	Manual mixing	[29]
	Termira AuxiGel™ (cross linked HA + PVA) + hydroxyapatite nanoparticles(nHA) + ZOL	Mandible	Commercial gel with ZOL Manual mixing; direct in situ injection	[15]
	Sodium titanate hydrogel	Mandible	Alkaline treatment	[26]
	Poloxamer 407 + β-TCP	Distal femur	Manual mixing	[30]
	GelMA/SilMA	Epithelial sealing in the peri-implant region	Photo-cross-linking (UV)	[8]
	HA-pNIPAM + BMP2 + ZOL	Proximal tibia	Thermal	[3]

Abbreviations: HPMC, hydroxypropyl methylcellulose; Si-HPMC, silanized hydroxypropyl methylcellulose; ZOL, Zoledronic acid; PEG, polyethylene glycol; BMP-2, Bone Morphogenetic Protein-2; PEG-SH4-4, arm polyethylene glycol with terminal thiol; rhBMP-2, Recombinant human bone morphogenetic protein-2; SEMA3A, Semaphorin-3A; GMP, Gelatin-Methacryloyl-Polypyrrole; OHA, Oxidized Hyaluronic Acid; SBS, Synthetic biphasic calcium phosphate; RGD, Arginine-Glycine-Aspartic acid; PVA, polyvinyl acid; Nha, hydroxyapatite nanoparticles; β-TCP-β, tricalcium phosphate; GelMA, Gelatin methacryloyl; SilMA, Silk fibroin glycidyl methacrylate; pNIPAM, Poly(N-isopropylacrylamide); UV, ultraviolet.

**Table 2:** Hydrogel classifications in studies on peri-implant defect repair, highlighting in vitro and in vivo results and the main physicochemical parameters relevant to bone regeneration.

Author and Year	Performed Tests	Gelation Mechanism	Degradation Mechanism	Porosity	Mechanical Strength	Gelation Time
Struillou et al. (2013) [19]	In vivo (dogs)	Not described	Not described	Not specified	Not specified	Not specified
Kettenberger et al. (2017) [27]	In vitro + In vivo (rats)	Hydrogel scaffold preloaded (gelation process not detailed)	Passive erosion / sustained release	Not specified	Not specified	Not specified
Jung et al. (2015) [22]	In vivo (dogs)	Crosslinking of PEG-hydrogel	Hydrolytic degradation	Low (unstable alone)	Low	Not specified
Pan et al. (2015) [28]	In vitro + In vivo (rats)	Spontaneous in situ gelation	Biodegradable natural polymer (not detailed)	High (sponge-like)	Elastic, adaptable	~1–5 min
Deng et al. (2023) [2]	In vitro + In vivo (rats)	“Click chemistry” in situ gelation	Enzymatic and hydrolytic (implied)	Not specified	Not specified	Fast
Qin et al. (2024) [24]	In vivo (rats)	Conductive hydrogel responsive to electrical microenvironment	Bioabsorbable (general description)	Not specified	Not specified	Not specified
Cha et al. (2018) [25]	In vivo (dogs)	Not described	Not described	Not described	Not described	Not specified
Thoma et al. (2017) [16]	In vivo (dogs)	In situ gelation of PEG (±RGD)	Hydrolytic degradation of PEG	Described only as porous	Sufficient for clinical use	Not specified
Seo et al. (2018) [29]	In vivo (dogs)	In situ polymerization	Diffusion and hydrolysis	Described only as porous	Maintains vertical support	Fast
Kettenberger et al. (2015) [15]	In vitro	Not described	Enzymatic degradation	Not specified	Not described	Fast
Kang et al. (2024) [26]	In vitro	Injectable peptide-functionalized hydrogel (mechanism not described)	Enzymatic degradation	Not described	Not described	Fast
Lee et al. (2014) [30]	In vivo (rabbits)	In situ gelation	Not described	Porosity of 68.5%	Not specified	Not specified
Li et al. (2022) [8]	In vivo (mice)	Photopolymerization	Biodegradable (not detailed)	Not described	Not described	Finished 60 s under 365 nm UV at 100 mW/cm^2^
Siverino et al. (2024) [3]	In vivo (rats)	Genipin-mediated chemical crosslinking	Slow degradation (not specified)	Porosity of ~68%	Maintains vertical support	Not described

Abbreviations: PEG, polyethylene glycol; BMP-2, Bone Morphogenetic Protein-2; HA-PEGDA, Hyaluronic Acid + Polietilenoglicol Diacrilato (Polyethylene Glycol Diacrylate); RGD, Arginine-Glycine-Aspartic Acid; UV, ultraviolet; nm, nanometer; mW/cm^2^, milliwatts per square centimeter.

### 3.3. Essential Properties of Hydrogels for Bone Regeneration

Among the fundamental properties for the success of hydrogels in peri-implant bone regeneration, the following stand out: biocompatibility, moldability to the bone defect, adequate mechanical stability, controlled biodegradability, porosity that allows cell infiltration and proliferation, as well as the ability to promote sustained release of bioactive agents. These characteristics are crucial for creating a microenvironment conducive to bone neoformation and osseointegration.

Studies such as those by Jung et al. [22] and Thoma et al. [16] indicate that polyethylene glycol (PEG) hydrogels, especially when combined with bone substitutes such as hydroxyapatite/calcium triphosphate (HA/TCP), contribute to bone regeneration in peri-implant defects, although their isolated mechanical stability may be limited, requiring additional support from particulate biomaterials. The addition of bioactive sequences such as RGD (Arginine: R, Glycine: G, and Aspartic Acid: D) to PEG hydrogels has also been shown to improve cell adhesion and tissue integration [22].

Similarly, Lee et al. [30] developed a composite of β-triphosphate calcium (β-TCP) with hydrogel as a carrier of BMP-2, which promoted greater bone formation around implants in an animal model, with a significant increase in trabecular thickness and bone-implant contact. Adequate porosity of the hydrogel was decisive for the penetration of the material into the peri-implant area and for cell infiltration.

Seo et al. [29] also demonstrated that an injectable poly(phosphazene) hydrogel with sustained release of BMP-2 resulted in vertical bone regeneration in critical defects, with a significant increase in bone mineral density and bone–implant contact rate, reinforcing the importance of the combination of bioactive release and structural support.

In addition, Struillou et al. [19] demonstrated that the association of hydroxypropylmethyl cellulose (HPMC) hydrogels with dicalcium phosphate improves graft stability and favors bone formation in dehiscence-type defects, including promoting direct bone-implant contact, with greater ease of handling during the surgical procedure and clot stability.

Therefore, the rational design of hydrogels applied to bone regeneration should consider not only their chemical composition, but also structural and functional aspects that allow for the integration of mechanical, biological, and pharmacokinetic properties, creating an environment that favors both osteoconduction and immune modulation, favorable to regeneration.

### 3.4. Structural and Functional Properties of Hydrogels in Peri-Implant Tissue Engineering

The physicochemical properties of hydrogels, determined primarily by the composition of the natural or synthetic polymers used, directly impact their applicability in tissue engineering aimed at peri-implant regeneration. Hydrogels based on natural polymers, such as hyaluronic acid or alginate, exhibit excellent biocompatibility and mimicry of the extracellular matrix, favoring cellular processes such as migration, adhesion, and differentiation [27,28,29]. However, these materials generally have lower mechanical strength and less precise control of manipulation kinetics, which may limit their stability in bone microenvironments with structural specifications. On the other hand, synthetic polymers, such as PEG or PCL, allow for greater customization of mechanical properties and the release of bioactive factors through varying crosslinking density and functionalization with biomolecules, such as RGD peptides [16,25], optimizing cell adhesion and promoting more controlled osteogenesis.

Gelification mechanisms, such as photoinitiation [30], mechanical addition [2], or in situ thermoregulation [29], directly modulate the morphology, porosity, and structural strength of hydrogels. Hydrogels with a porous structure favor vascular infiltration and nutrient diffusion, essential for bone formation around implants [24,29]. Controlled manipulation kinetics are particularly relevant in this context, as they must ensure the integrity of the scaffold during the initial phases of osseointegration, enabling progressive load transfer to the newly formed bone tissue.

Thus, while natural hydrogels offer advantages in terms of bioactivity and cellular interaction, synthetic hydrogels offer greater variety to meet the biomechanical demands of peri-implant regeneration. The rational choice between these materials, or their combination in hybrid systems, must consider the characteristics of the peri-implant bone defect, the healing stage, and local biomechanical stimuli, in order to improve bone regeneration and long-term implant integration.

### 3.5. Hydrogels and Their Immunomodulatory and Pro-Angiogenic Properties

Recent studies demonstrate that certain hydrogels, such as PEG derivatives, hyaluronic acid (HA), and in situ polymerizable systems via click chemistry, can be functionalized to simultaneously promote immunomodulatory and pro-angiogenic effects [2,8,28]. The local and controlled release of biomolecules, such as BMP-2 and zoledronic acid, is effective in attenuating exacerbated inflammation and stimulating cellular and vascular recruitment, processes fundamental for osseointegration in conditions of poor bone quality or systemic impairment, as occurs in [3,22,25].

At the same time, the incorporation of cell adhesion peptides (such as RGD), conductive materials, or osteoconductive ceramic particles has been associated with early angiogenesis induction and macrophage polarization toward the M2 phenotype, favoring a more efficient regenerative microenvironment [8,24]. Among the bioactive agents studied, semaphorin 3A stands out for its action in both modulating the inflammatory response and promoting vascularization, contributing decisively to successful osseointegration [2].

Furthermore, the combination of hydrogels with bioactive ceramics or functional peptides [16,19] enhances bone regeneration by providing graft stability, initial mechanical support, and stimulating vascular infiltration. This integrated approach has proven particularly effective in challenging clinical contexts, such as in diabetic patients or those with severe bone loss, in which the balance between immunoregulation, revascularization, and osteogenesis is crucial for successful implant integration [3,15].

### 3.6. Hydrogels as Biomolecule Delivery Systems

One of the main functions of hydrogels in bone tissue engineering is to act as controlled release systems for osteoinductive factors, such as BMP-2, sema3A, bisphosphonates, or simvastatin. The use of hydrogels as carriers allows for a reduction in the required dose of the bioactive factor, minimizing adverse effects and prolonging the time of action, providing a favorable microenvironment for bone regeneration. Studies demonstrate that the incorporation of BMP-2 into hydrogels can significantly increase peri-implant bone formation, including critical bone defect models [21,25,29]. Compared to conventional methods, Pan et al. [28] showed that the sustained release of rhBMP-2 from hyaluronic acid hydrogels optimized new bone formation around canine mandibular implants.

In addition to BMP-2, other therapeutic agents have been tested in combination with hydrogels. Siverino et al. [3] showed that local delivery of BMP-2 and zoledronic acid by thermoresponsive hydrogel significantly improved osseointegration in osteoporotic bone. Similarly, Kettenberger et al. [15] demonstrated that the combination of hydroxyapatite with bisphosphonate-containing hyaluronic acid promoted rapid mineralization and inhibition of bone resorption around implants in an osteoporotic model, increasing peri-implant bone density. The incorporation of hydroxyapatite (nHA) nanoparticles and zoledronic acid into hyaluronic acid hydrogel has demonstrated potential to promote peri-implant bone repair, promoting a more stable and bioactive microenvironment for tissue regeneration [27].

Deng et al. [2] introduced an innovative approach using semaphorin 3A (sema3A), a neurogenic factor with osteoprotective properties, transported by a rapidly cross-linked polyethylene glycol hydrogel. This strategy significantly restored osseointegration in type 2 diabetic models, a challenging clinical condition due to poor bone quality. These data reinforce the potential of hydrogels as versatile and intelligent platforms for the delivery of multiple osteoinductive agents, with promising application in patients with systemic conditions, and adverse effects on bone regeneration [2].

It is important to highlight that the efficacy of the hydrogel depends strongly on its physicochemical properties, such as biocompatibility, mechanical stability, release kinetics, and defect-filling capacity. Jung et al. [22] and Thoma et al. [16] reinforce that, although PEG hydrogel has advantages in the release of factors such as BMP-2, its structural stability may be limited, being more effective when combined with particulate bone substitutes, such as hydroxyapatite/calcium triphosphate (HA/TCP).

Finally, Struillou et al. [19] demonstrated that the association of hydrogel with dicalcium phosphate in dehiscence-type peri-implant defects significantly improved graft stability and favored bone–implant contact, indicating a viable alternative to autogenous grafts in complex clinical situations.

### 3.7. Applications in Experimental and Clinical Models

The use of hydrogels in the peri-implant setting has been extensively investigated in both preclinical studies and clinical trials, reflecting their translational potential in regenerative and therapeutic dentistry. Preclinical applications in vitro models provide fundamental data on the material’s predictive potential and its biological performance under controlled conditions [8,27]. Furthermore, animal models (such as rats, mice, and rabbits) have been used to simulate peri-implant pathologies and critical bone defects, allowing for an investigation of the efficacy of hydrogels in tissue regeneration, modulation of the inflammatory response, and infection control [8].

For example, studies such as those by Jung et al. [22], Thoma et al. [16], and Cha et al. [25] demonstrated the formation of new bone tissue around implants with the use of bioactive hydrogels containing osteoinductive factors, such as BMP-2, and to improve adhesion, proliferation, and osteogenic differentiation, such as the use of arginylglycylaspartic acid (RGD). In challenging conditions, such as in type 2 diabetes models, Deng et al. [2] showed that the local application of Semaphorin 3A, a hydrogel based on “click” chemistry, contributed to the osseointegration of implants.

Kang et al. [26] showed that surface treatments with bioactive titanate hydrogels increased in vitro bioactivity and stimulated cell adhesion and proliferation, representing a promising strategy for improving osseointegration in dental implants.

Although the clinical use of hydrogels in humans is still in its early stages, the available results are promising, especially for minimally invasive and personalized approaches. However, the literature still lacks robust clinical studies that validate the long-term efficacy and safety of these applications.

Despite the existence of clinical studies on the use of hydrogels, during the screening and selection process of articles specifically related to the application of these biomaterials in peri-implant defects, no clinical studies met the inclusion criteria defined for this review, reinforcing the need for further clinical investigations focused on this context.

### 3.8. Current Challenges and Future Perspectives

Despite significant advances in tissue engineering, challenges persist in the clinical translation of hydrogels. Among the main obstacles are mechanical stability in large (three-dimensional) defects, precise control of degradation, and localized and sustained release of biomolecules, such as growth factors. In addition, there is an urgent need to standardize experimental models and develop formulations that are easy to apply clinically, especially in complex surgical environments [21,22].

Studies such as that by Jung et al. [22] have demonstrated that polyethylene glycol (PEG)-based hydrogels, when loaded with BMP-2, are biocompatible and promote peri-implant bone formation even in critical defects, although they present limitations regarding isolated structural stability. Thoma et al. [16] observed that the addition of bioactive sequences such as RGD to PEG can improve cell adhesion and stimulate bone regeneration, but the results were inferior when compared to conventional guided regeneration techniques.

Other innovative strategies include the use of hydrogels with sustained release of bioactive proteins, as demonstrated by Seo et al. [29], where a poly(phosphazene) hydrogel with controlled release of BMP-2 led to significant vertical bone regeneration in a canine model.

The future of the area points to smart hydrogels, capable of responding to biological stimuli (such as pH, enzymes or temperature), associated with the integration of stem cells and the use of technologies such as 3D printing for the construction of personalized grafts, with the potential to transform regenerative medicine into environments conducive to peri-implant bone regeneration [2,15,29].

To date, the few existing trials have involved experimental applications or adjuvant treatments in limited settings. Therefore, there is a clear need for more well-controlled clinical research that evaluates parameters such as osseointegration rate, implant stability, immunological response, and economic viability, especially in personalized and minimally invasive settings.

## 4. Discussion

Although peri-implant bone regeneration has advanced, complex clinical cases, such as poor bone quality, low vascularization, and the presence of systemic comorbidities, still require more effective approaches [31,32]. In this context, hydrogels have emerged as promising bioactive platforms capable of modulating biological responses, promoting implant integration, and offering tunable properties, such as controlled release of biomolecules. These characteristics not only facilitate overcoming regenerative barriers but also personalizing treatment according to the pathological microenvironment [33]. Furthermore, they represent a viable alternative to autogenous grafts, which have significant limitations, such as donor site morbidity, risk of contamination, and limited availability [34]. Therefore, advances in hydrogel formulations and functionalities directly respond to clinical demands.

The comparative efficacy of natural and synthetic hydrogels is still debated in the literature, in part due to the lack of experimental standardization. Natural hydrogels, such as those derived from hyaluronic acid or cellulose, exhibit high biocompatibility, better replicate the extracellular matrix, and promote favorable biological interactions [35]. However, they tend to have lower mechanical strength and unpredictable degradation. Synthetic hydrogels, such as those derived from polyethylene glycol (PEG), allow precise control over physicochemical properties and drug release kinetics, in addition to exhibiting greater structural stability, although they require biofunctionalization to induce adequate cellular responses [36,37]. In this context, hybrid systems, which combine PEG with osteoconductive ceramics such as hydroxyapatite or β-TCP, seek to combine the benefits of both types, presenting better clot stability and greater bone–implant contact in animal models, reinforcing their translational potential [38,39].

The success of peri-implant bone regeneration is intrinsically linked to the ability of biomaterials to modulate the immune microenvironment and promote efficient angiogenesis [40]. Modern hydrogels have been designed to actively address these issues, integrating the controlled release of immunomodulatory biomolecules (such as semaphorin 3A or zoledronic acid) and pro-angiogenic biomolecules (such as VEGF or BMP-2), as well as the incorporation of cell adhesion peptides and conductive particles [41]. This multifunctional approach has been shown to reduce the initial exacerbation of inflammation and promote the formation of new blood vessels, essential conditions for cellular infiltration and bone matrix deposition. The convergence of these mechanisms has been particularly promising in models of poor bone quality, such as osteoporosis or diabetic patients, highlighting the relevance of regenerative strategies that simultaneously act on the immune and vascular systems [41].

Functionalizing hydrogels with bioactive agents is one alternative to enhance their properties. The incorporation of osteoinductive factors (BMP-2), immunomodulatory molecules (semaphorin 3A), osteoprotective drugs (bisphosphonates), and bioactive peptides (RGD motifs) has been shown to increase cell adhesion, osseointegration, and bone regeneration [2,42,43,44]. Furthermore, advances in crosslinking techniques, including Michael addition, enzymatic reactions, and photoinduced reactions (GelMA), allow for the fine-tuning of mechanical properties, degradation rates, and controlled release of bioactives [45]. Therefore, the development of stimuli-responsive hydrogels that adapt to peri-implant microenvironmental changes represents a promising innovation.

Functionalized hydrogels loaded with bioactive molecules or stem cells have demonstrated the ability to reduce chronic inflammation and enhance tissue remodeling [46,47]. Innovative multiphase or sequential-release hydrogel platforms allow the temporal delivery of biomolecules aligned with distinct stages of bone healing, for example, early release of vascular endothelial growth factor to stimulate neovascularization, followed by delayed administration of BMP-2 to promote osteogenesis [44,48,49,50]. Furthermore, hydrogels have the potential to improve soft tissue sealing around implants, thus reducing bacterial infiltration and the risk of peri-implantitis [51,52]. Their application as barrier membranes or antimicrobial delivery systems further reinforces their multifunctionality in implant dentistry.

The literature on hydrogels in peri-implant bone repair gathers robust evidence from in vitro and in vivo models, and, to a lesser extent, clinical studies [3,53]. Studies with conductive hydrogels demonstrated in vitro activation of pro-osteogenic signaling pathways via intracellular Ca^2+^, and in vivo increased expression of RUNX2 and osteocalcin in peri-implant areas [24]. Furthermore, they actively modulate the immune response, promoting the polarization of macrophages toward the anti-inflammatory M2 phenotype, thus fostering a regenerative environment [54]. Clinical studies, although still limited, have reported benefits such as reduced bone resorption and greater primary stability in guided regeneration procedures [3]. The triangulation of these approaches strengthens the scientific basis for hydrogels, although it highlights the need for more controlled, long-term clinical trials.

Compared to traditional materials used in bone regeneration, such as hydroxyapatite, β-TCP, or bioactive glasses, hydrogels offer distinct advantages that make them attractive for periodontal and peri-implant applications [55,56]. Their high porosity and water content provide a three-dimensional environment favorable to nutrient transport, cell migration, and the exchange of biochemical signals [56]. Furthermore, the ability to be functionalized with bioactive molecules and release growth factors in a controlled manner gives hydrogels superior therapeutic potential, enabling more specific and customizable responses [3,56,57]. While biomaterials offer excellent osteoconductivity, newer hydrogels can also promote osteoinduction and modulate immune responses, filling gaps that conventional materials do not fully address [57].

Although bioactive hydrogels have shown promising results in preclinical studies, their clinical application remains limited. Factors such as variability between animal and human models, a lack of standardized protocols, stringent regulatory requirements (such as Good Manufacturing Practices(GMPs)), and difficulties in scalability hinder their transition to clinical practice. Furthermore, challenges related to mechanical stability, degradation control, and localized release of bioactives persist, as do economic barriers that limit their adoption compared to established biomaterials [58].

Future directions emphasize the development of smart hydrogels responsive to the endogenous environment, enabling personalized and sequential release of bioactives. Integration with emerging technologies, such as three-dimensional bioprinting, offers promising potential for the fabrication of personalized grafts tailored to the anatomy and specific needs of the patient [59]. Randomized clinical trials with long-term follow-up are crucial to confirm safety, efficacy, and cost-effectiveness, and to drive the integration of these technologies into routine clinical practice [3,58].

## 5. Conclusions

Based on current literature, hydrogels emerge as promising platforms for peri-implant bone regeneration, thanks to their versatility, biocompatibility, and functionalization potential. Despite preclinical advances, clinical application still requires overcoming technical, regulatory, and economic barriers. Strategies that integrate controlled release of bioactives, immune modulation, and angiogenesis are essential to enhance their efficacy. The development of smart and personalized hydrogels, combined with robust clinical trials, will be crucial to consolidating their application in dental practice.

## Data Availability

Not applicable.
